# Data on characterization of nano- and micro-structures resulting from glycine betaine surfactant/kappa-carrageenan interactions by Laser Scanning Confocal Microscopy and Transmission Electron Microscopy

**DOI:** 10.1016/j.dib.2016.09.026

**Published:** 2016-09-22

**Authors:** Cédric Gaillard, Yunhui Wang, Rudy Covis, Thomas Vives, Maud Benoit, Thierry Benvegnu

**Affiliations:** a U.R. 1268 Biopolymères Interactions Assemblages INRA BP-71, 627 Rue de la Géraudière, 44316 Nantes Cedex 3, France; b Ecole Nationale Supérieure de Chimie de Rennes, CNRS UMR6226, 11 allée de Beaulieu, CS50837, 35708 Rennes Cedex 7, France; c Université de Bretagne Loire, France; d Centre d׳étude et de Valorisation des Algues, Presqu׳île de Pen Lan – BP3, 22610 Pleubian, France

**Keywords:** Glycine betaine surfactant/kappa-carrageenan complexes, Nano- and micro-structures, Electrostatic interactions, Laser Scanning Confocal Microscopy and Transmission Electron Microscopy

## Abstract

This article contains data on the Laser Scanning Confocal Microscopy (LSCM) and Transmission Electron Microscopy (TEM) images related to multi-scaled self-assemblies resulting from ‘green’ cationic glycine betaine surfactant/anionic kappa-carrageenan interactions. These data gave clear evidence of the evolution of the micron-, nano-sized structures obtained at two surfactant/polymer molar ratios (3.5 and 0.8) and after the dilution of the aqueous dispersions with factors of 5 and 10 times. This data article is related to the research article entitled, “Monitoring the architecture of anionic ĸ-carrageenan/cationic glycine betaine amide surfactant assemblies by dilution: A multiscale approach” (Gaillard et al., 2017) [1].

**Specifications Table**

**Table:** 

Subject area	*Chemistry, Material Sciences, Soft Matter*
More specific subject area	*Structural analysis of nano-, micro- structures*
Type of data	*Figures*
How data was acquired	*Laser Scanning Confocal Microscopy (LSCM, Inverted Nikon A1 laser scanning confocal microscope (LSCM) and Transmission Electron Microscopy (TEM, JEOL JEM-1230 operated at 80 kV and equipped with a LaB6 filament*
Data format	*Analyzed*
Experimental factors	*LSCM: Aqueous dispersions of the surfactant/polysaccharide complexes were stained with 0.02% w/w acridine orange*
	*TEM: Sample-coated TEM grid was successively placed on a drop of an aqueous solution of uranyl acetate (2% w/w) for negatively staining, and on a drop of distilled water for rinsing. The grid was then air-dried before introducing them in the electron microscope*
Experimental features	*LSCM: samples viewed with Plan Fluor 4× or 10× Nikon objectives or with Plan Apo 20× or 40× Nikon objective by scanning using excitations brought about by the 488 nm emission and 561 nm emission lines of the He–Ne laser, and light emission was collected via a photomultiplier through a 500–530 nm and 570–620 nm band-pass filters, respectively. Images were processed using the NIS-Element*
	*TEM: micrographs were recorded on a Gatan 1.35 K×1.04 K×12 bit ES500W CCD camera.*
Data source location	*U.R. 1268 Biopolymères Interactions Assemblages INRA BP-71, 627 Rue de la Géraudière, 44316 Nantes Cedex 3, France*
Data accessibility	*Data is with this article*

**Value of the data**

• The given data provide structural information of particles based on multi-components at the micron- and nanometer scale range by using Laser Scanning Confocal Microscopy (LSCM) [2], [3], [4], and Transmission Electron Microscopy (TEM).
• The data provided by us help to understand the mechanism of formation of self-assemblies resulting from electrostatic interactions between multi-components.
• The data provided by us show influence of dilution on the architecture of assemblies composed of anionic polymers/cationic surfactants derived from renewable resources.
• The given data are useful to other researchers for developing applications of multi-scaled self-assemblies by mixing simply polymers and surfactants of opposite charge.

## 1 Data

**Fig. 1:**
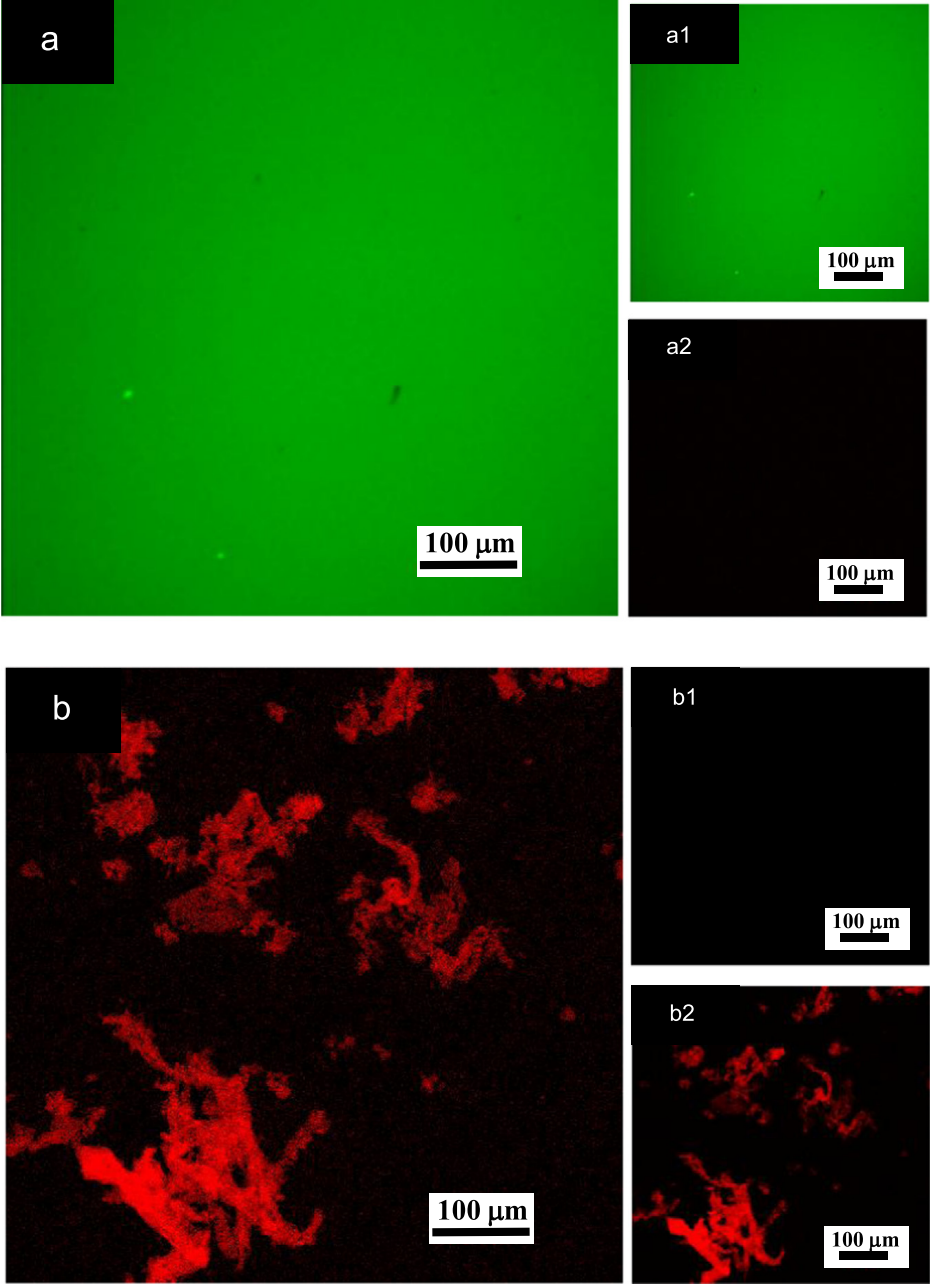
LSCM images of aqueous solutions of (a) pure surfactant (10 g/L) and (b) pure ĸ-carrageenan (10 g/L) after fluorescence staining with acridine orange (0.02 % v/v). (a)–(b): LSCM green and red merged canals for both surfactant and ĸ-carrageenan emissions; (a1)–(b1): LSCM green canal corresponding to surfactant emission at 500–530 nm; (a2)–(b2): LSCM red canal corresponding to ĸ-carrageenan emission at 570–620 nm.

**Fig. 2:**
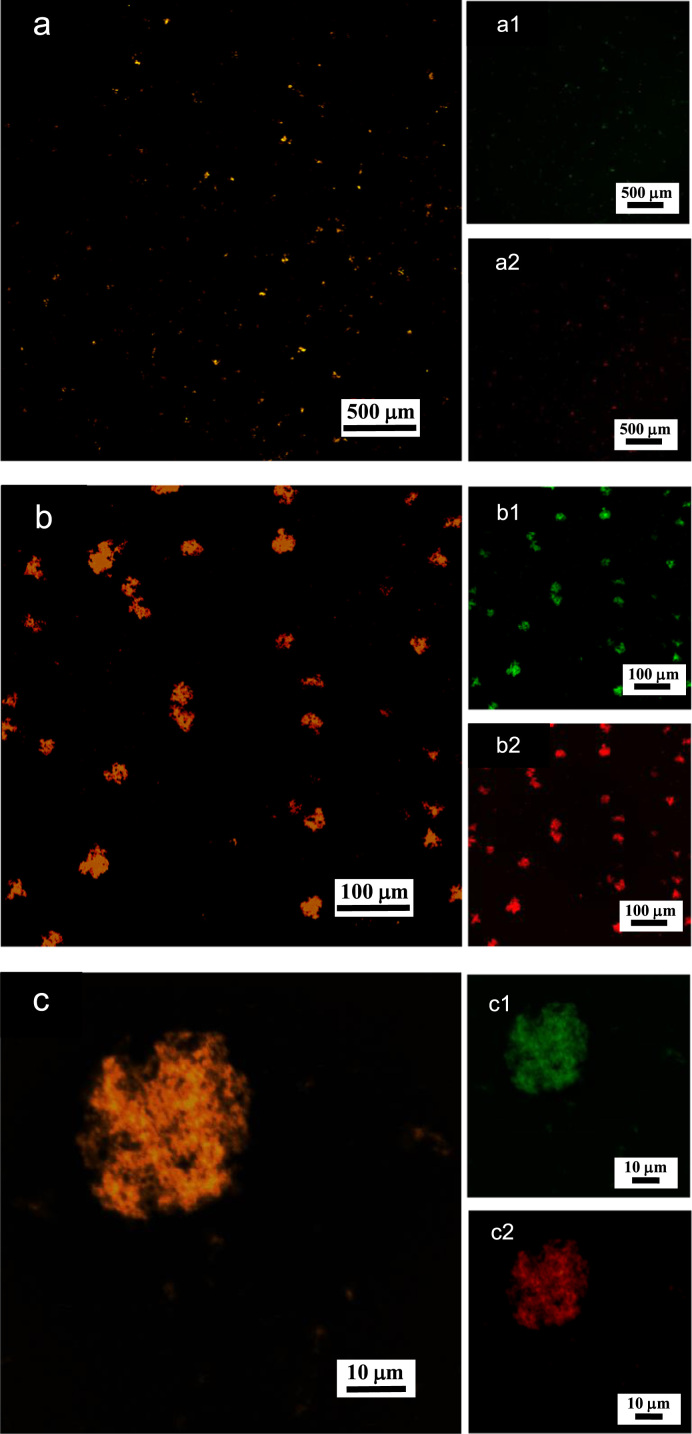
LSCM images of aqueous dispersions (Sample (**A1**)) containing ĸ-carrageenan at a concentration of 0.825 g/L and surfactant at a concentration of 2.9 g/L. Sample (**A1**) was stained with acridine orange for which the surfactant and ĸ-carrageenan emissions correspond to 500–530 nm (green canal) for an excitation of 488 nm, and 570–620 nm (red canal) for an excitation of 561 nm, respectively. (a)–(c): LSCM green and red merged canals for both surfactant and ĸ-carrageenan emissions; (a1)–(c1): LSCM green canal corresponding to surfactant emission at 500–530 nm; (a2)–(c2): LSCM red canal corresponding to ĸ-carrageenan emission at 570–620 nm.

**Fig. 3:**
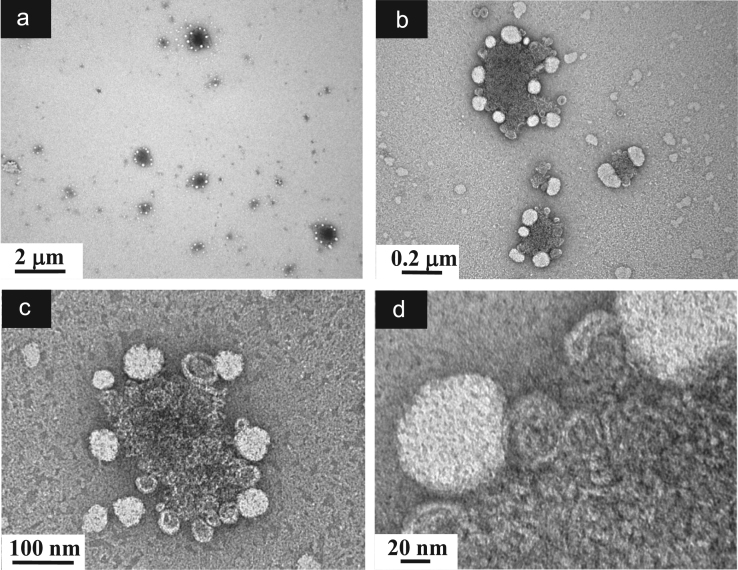
TEM images of aqueous dispersions (Sample (**A1**)) containing ĸ-carrageenan at a concentration of 0.825 g/L and surfactant at a concentration of 2.9 g/L. (a): Global view showing sub-micronsized particles; (b)–(c): Higher magnification views showing the singular morphology of the Sample (**A1**) particles described by a compact core decorated with peripheral spherical-liked regions; (d): Details of the outer part of a particle showing peripheral spherical surfactant rich regions connected to rolled up ĸ-carrageenan chains located in the particle center.

**Fig. 4:**
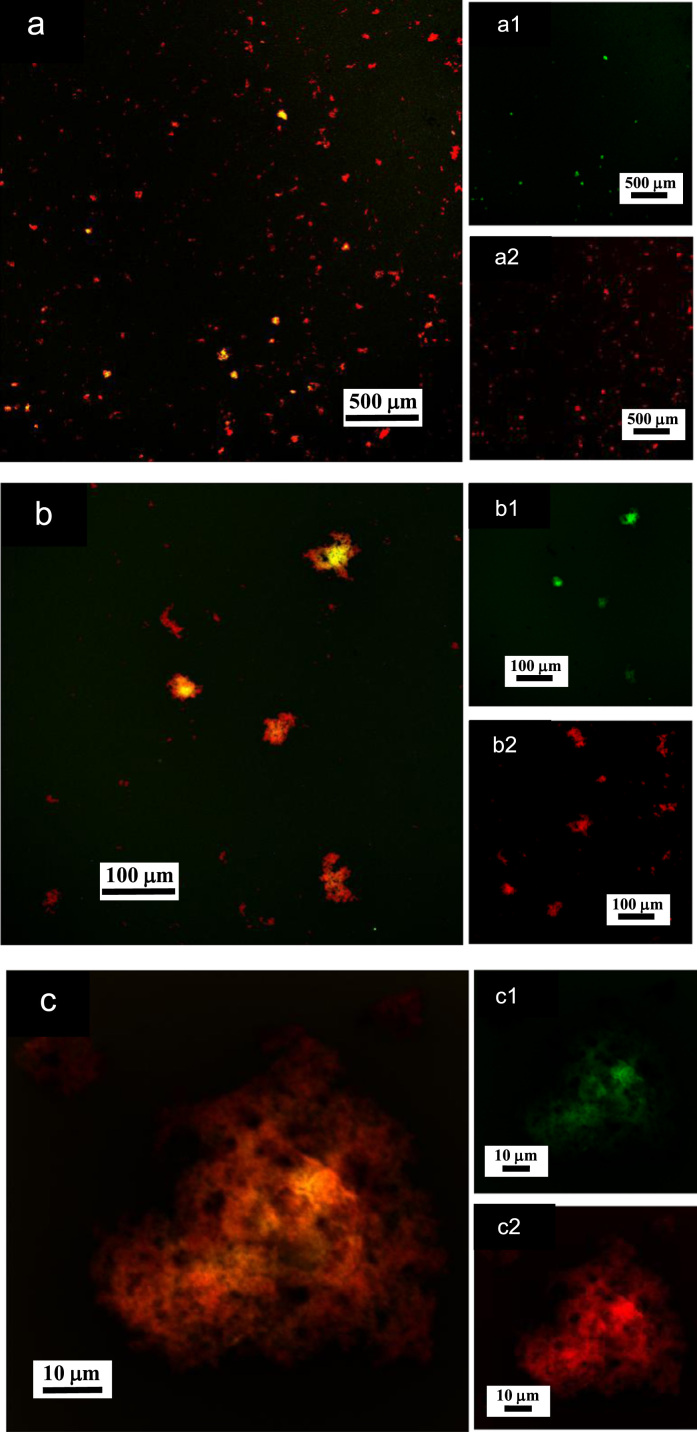
LSCM images of aqueous dispersions (Sample (**A2**)) containing ĸ-carrageenan at a concentration of 0.165 g/L and surfactant at a concentration of 0.58 g/L. Sample (**A2**) was stained with acridine orange for which the surfactant and ĸ-carrageenan emissions correspond to 500–530 nm (green canal) for an excitation of 488 nm, and 570–620 nm (red canal) for an excitation of 561 nm, respectively. (a)–(c): LSCM green and red merged canals for both surfactant and ĸ-carrageenan emissions; (a1)–(c1): LSCM green canal corresponding to surfactant emission at 500–530 nm; (a2)–(c2): LSCM red canal corresponding to ĸ-carrageenan emission at 570–620 nm.

**Fig. 5:**
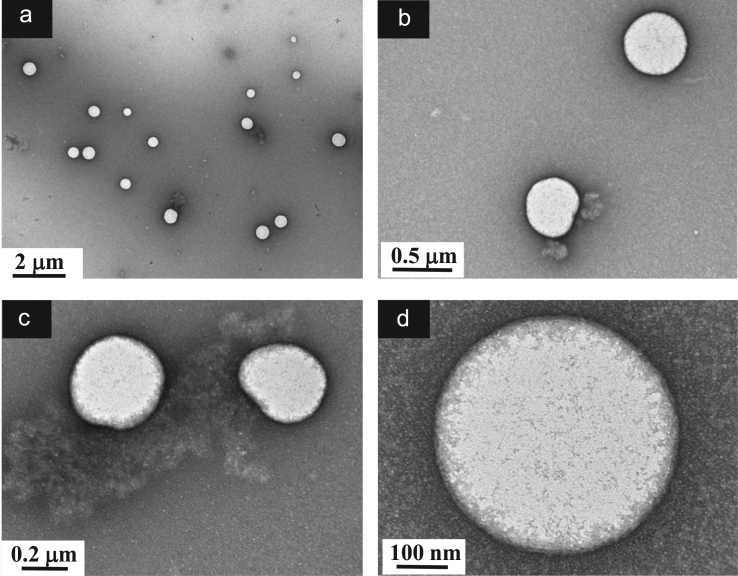
TEM images of aqueous dispersions (Sample (**A2**)) containing ĸ-carrageenan at a concentration of 0.165 g/L and surfactant at a concentration of 0.58 g/L. (a): Global view showing sub-micronsized polymer-surfactant complexes; (b)–(c): Higher magnification views showing the singular morphology of the Sample (**A2**) particles; (d): Details of a spherical-liked particle attributed to surfactant rich zones of the complexes.

**Fig. 6:**
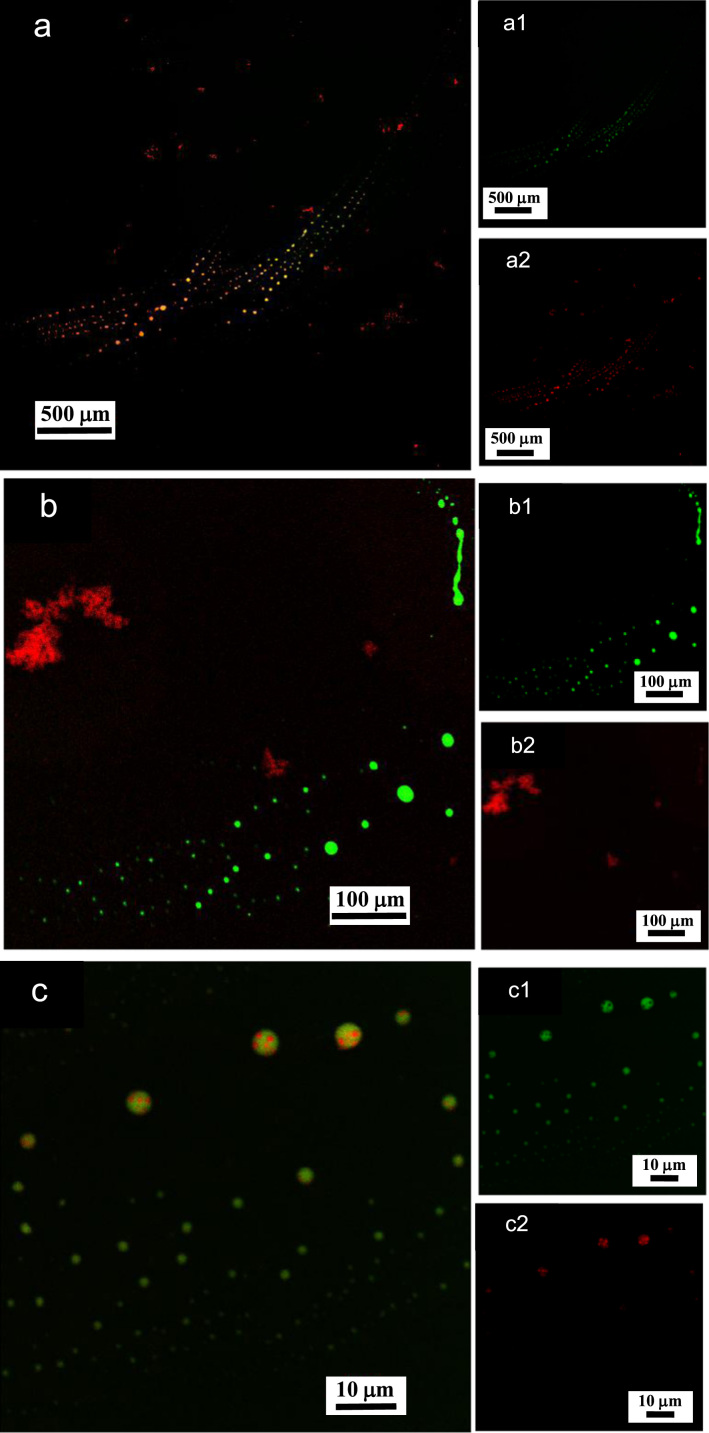
LSCM images of aqueous dispersions (Sample (**A3**)) containing ĸ-carrageenan at a concentration of 0.0825 g/L and surfactant at a concentration of 0.29 g/L. Sample (**A3**) was stained with acridine orange for which the surfactant and containing ĸ-carrageenan emissions correspond to 500–530 nm (green canal) for an excitation of 488 nm, and 570–620 nm (red canal) for an excitation of 561 nm, respectively. (a)–(c): LSCM green and red merged canals for both surfactant and containing ĸ-carrageenan emissions; (a1)–(c1): LSCM green canal corresponding to surfactant emission at 500–530 nm; (a2)–(c2): LSCM red canal corresponding to ĸ-carrageenan emission at 570–620 nm.

**Fig. 7:**
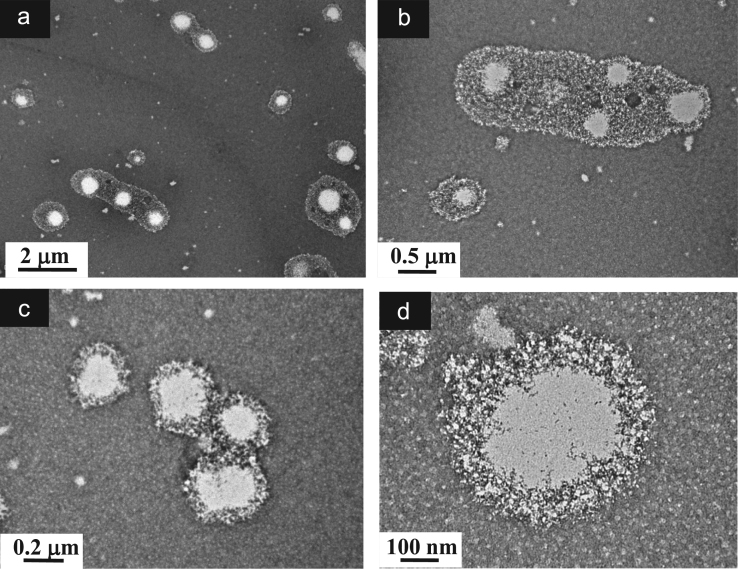
TEM images of aqueous dispersions (Sample (**A3**)) containing ĸ-carrageenan at a concentration of 0.0825 g/L and surfactant at a concentration of 0.29 g/L. (a): Global view showing the morphology of the sub-micronsized polymer-surfactant complexes; (b)–(c): Higher magnification views showing the singular morphology of the Sample (**A3**) particles designed by a dense core and a discontinuous shaped shell; (d): Details of a core–shell particle where the core and shell are attributed to surfactant and polymer, respectively.

**Fig. 8:**
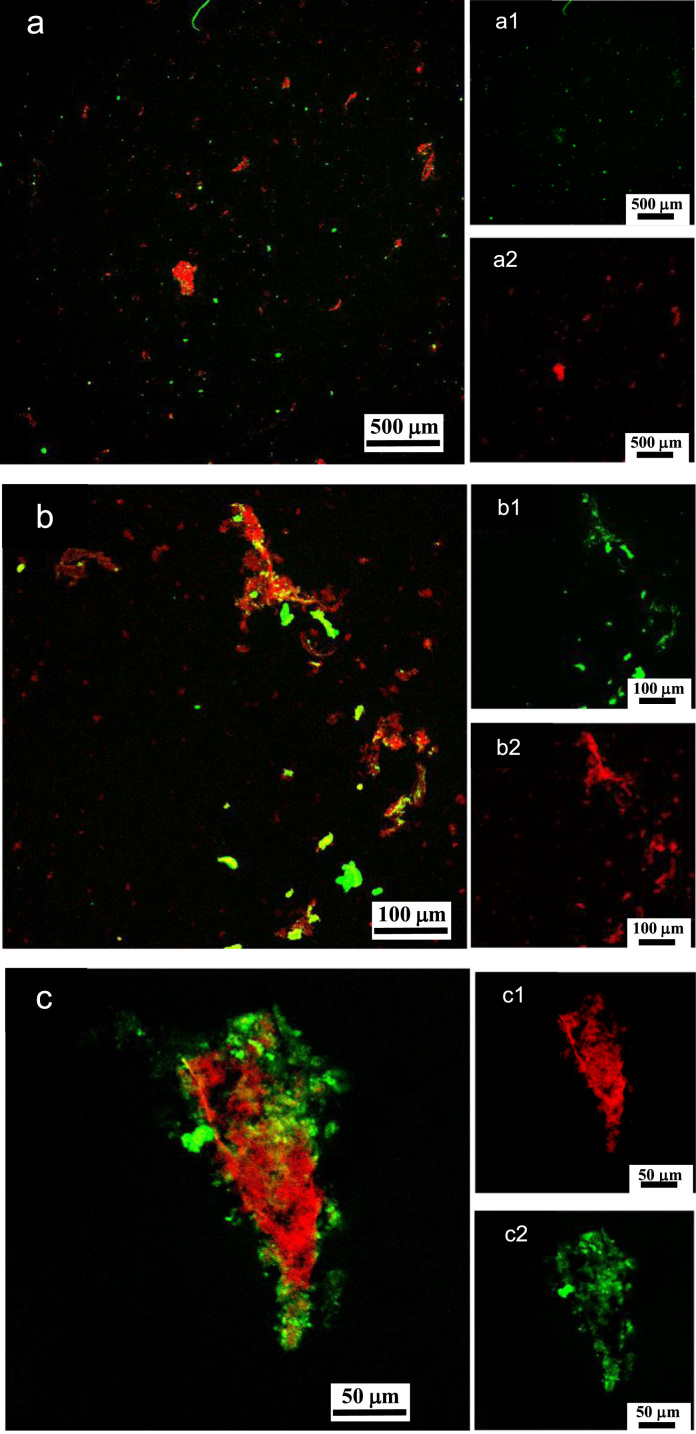
LSCM images of aqueous dispersions (Sample (**B1**)) containing ĸ-carrageenan at a concentration of 0.95 g/L and surfactant at a concentration of 0.83 g/L. Sample (**B1**) was stained with acridine orange for which the surfactant and ĸ-carrageenan emissions correspond to 500–530 nm (green canal) for an excitation of 488 nm, and 570–620 nm (red canal) for an excitation of 561 nm, respectively. (a)–(c): LSCM green and red merged canals for both surfactant and ĸ-carrageenan emissions; (a1)–(c1): LSCM green canal corresponding to surfactant emission at 500–530 nm; (a2)–(c2): LSCM red canal corresponding to ĸ-carrageenan emission at 570–620 nm.

**Fig. 9:**
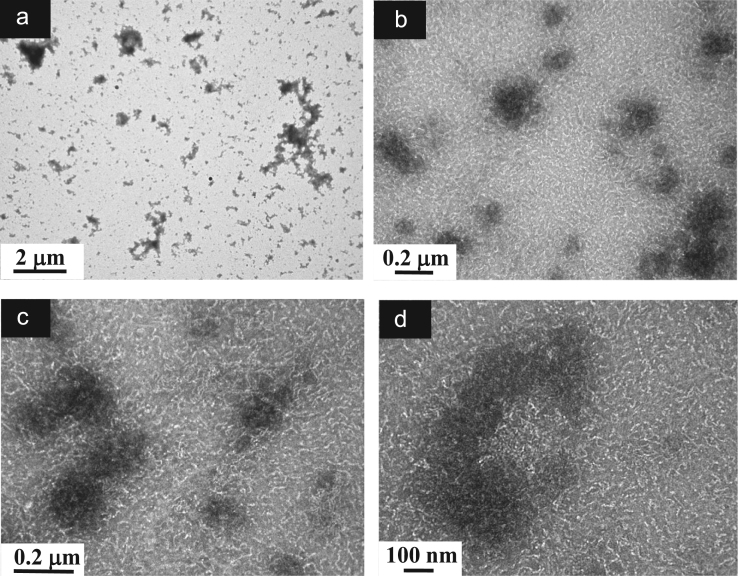
TEM images of aqueous dispersions (Sample (**B1**)) containing ĸ-carrageenan at a concentration of 0.95 g/L and surfactant at a concentration of 0.83 g/L. (a): Global view showing particles of various sizes and shapes; (b)–(c): Higher magnification views showing the morphology of the Sample (**B1**) particles constituted by sub-micronsized more or less associated dense particles and numerous individual short chains located on the background; (d): Details of the chains attributed to ĸ-carrageenans and taking different configurations due to a relative flexibility.

**Fig. 10:**
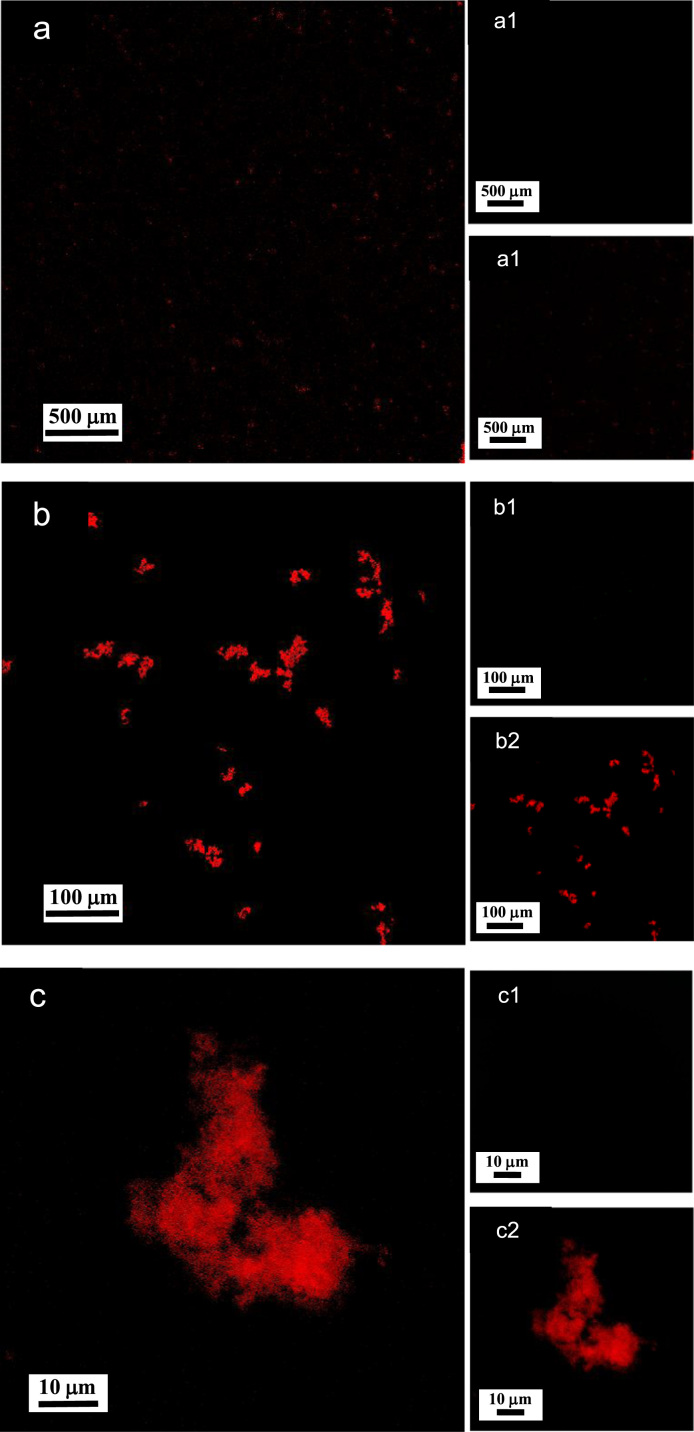
LSCM images of aqueous dispersions (Sample (**B2**)) ĸ-carrageenan at a concentration of 0.19 g/L and surfactant at a concentration of 0.166 g/L. Sample (**B2**) was stained with acridine orange for which the surfactant and ĸ-carrageenan emissions correspond to 500–530 nm (green canal) for an excitation of 488 nm, and 570–620 nm (red canal) for an excitation of 561 nm, respectively. (a)–(c): LSCM green and red merged canals for both surfactant and k-carrageenan emissions; (a1)–(c1): LSCM green canal corresponding to surfactant emission at 500–530 nm; (a2)–(c2): LSCM red canal corresponding to ĸ-carrageenan emission at 570–620 nm.

**Fig. 11:**
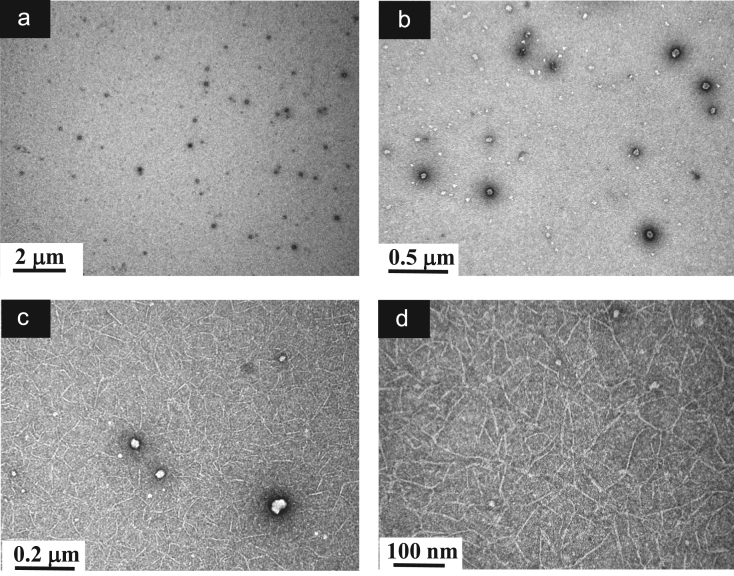
TEM images of aqueous dispersions (Sample (**B2**)) ĸ-carrageenan at a concentration of 0.19 g/L and surfactant at a concentration of 0.166 g/L. (a): Global view showing a distribution of nanoparticles; (b)–(c): Higher magnification views showing the morphology of the Sample (**B2**) particles constituted by spherical-liked nanoparticles and numerous individual long rod-liked chains located on the background; (d): Details of the long chains attributed to ĸ-carrageenans with long rigid segments leading to a network of percolated rods.

**Fig. 12:**
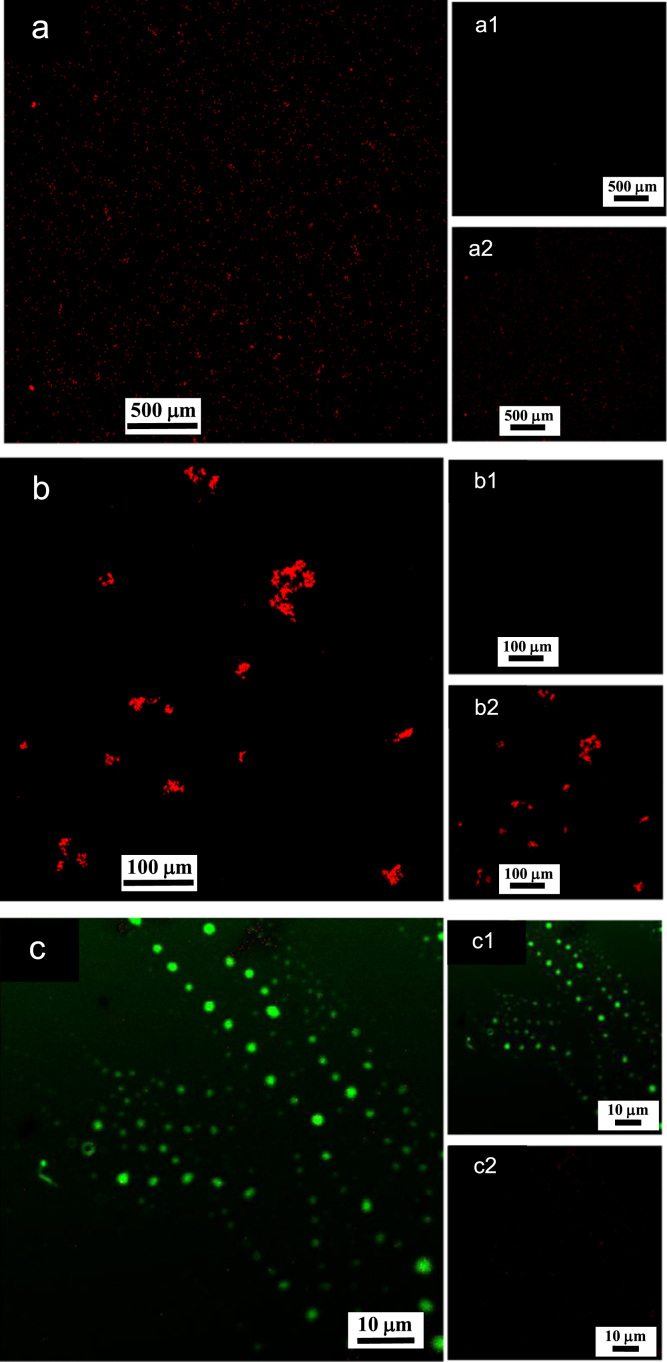
LSCM images of aqueous dispersions (Sample (**B3**)) containing ĸ-carrageenan at a concentration of 0.095 g/L and surfactant at a concentration of 0.083 g/L. Sample (**B3**) was stained with acridine orange for which the surfactant and ĸ-carrageenan emissions correspond to 500–530 nm (green canal) for an excitation of 488 nm, and 570–620 nm (red canal) for an excitation of 561 nm, respectively. (a)–(c): LSCM green and red merged canals for both surfactant and ĸ-carrageenan emissions; (a1)–(c1): LSCM green canal corresponding to surfactant emission at 500–530 nm; (a2)–(c2): LSCM red canal corresponding to ĸ-carrageenan emission at 570–620 nm.

**Fig. 13:**
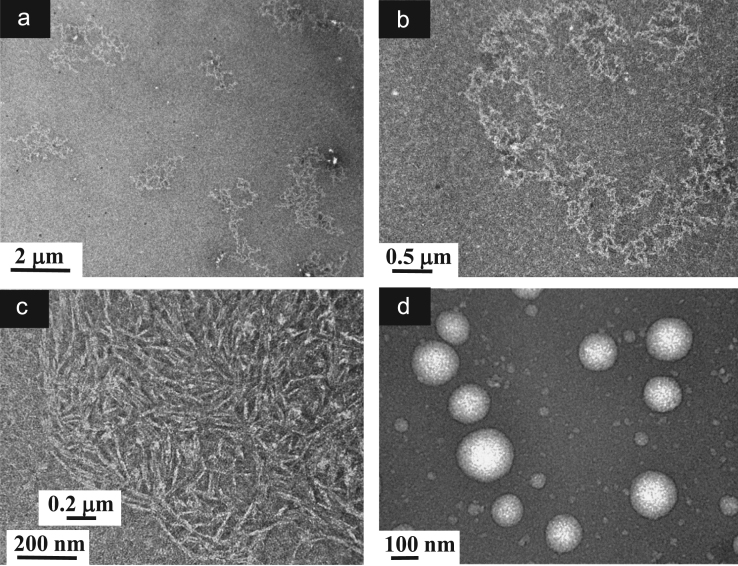
TEM images of aqueous dispersions (Sample (**B3**)) containing ĸ-carrageenan at a concentration of 0.095 g/L and surfactant at a concentration of 0.083 g/L. (a): Global view showing a distribution of aggregates resulting from k-carrageenan along with spherical-liked particles; (b)–(c): Higher magnification views of an aggregate connected to a spherical-like particle; (c) Details of aggregates formed by rolled-up ĸ-carrageenan; (d): Details of spherical-liked particles.

Data refers to the LSCM and TEM experiments of 100% bio-sourced glycine betaine (GB) surfactant possessing a C_18:1_ oleic fatty chain and kappa-carrageenan under pure forms in aqueous solutions (Fig. 1) or after their mixing at two different GB surfactant/κ-carrageenan molar ratios equal to *3.5* (sample A1: Fig. 2, Fig. 3) and *0.8* (sample B1: Fig. 8, Fig. 9) and after a dilution with a factor of 5 (*ratio 3.5* (sample A2): Fig. 4, Fig. 5; *ratio 0.8* (sample B2): Fig. 10, Fig. 11) and 10 (*ratio 3.5* (sample A3): Fig. 6, Fig. 7; *ratio 0.8* (sample B3): Fig. 12, Fig. 13) times. TEM observation shows the gradual dissociation of assemblies’ nanostructures whereas LSCM identifies the distribution of cationic surfactant and anionic polysaccharide.

## 2 Experimental design, materials and methods

Materials and Methods adopted LCSM and TEM experiments have been already described by us in our previously published article (http://dx.doi.org/10.1016/j.carbpol.2016.08.027) [1].

## References

[1] Gaillard C., Wang Y., Vives T., Benoit M., Benvegnu T. (2017). Monitoring the architecture of anionic ĸ-carrageenan/cationic glycine betaine amide surfactant assemblies by dilution: a multiscale approach. Carbohydr. Polym..

[2] Traganos F., Darzynkiewicz Z., Sharpless T., Melamed M.R. (1977). Simultaneous staining of ribonucleic and deoxyribonucleic acids in unfixed cells using acridine orange in a flow cytofluorometric system. J. Histochem. Cytochem..

[3] Rigler R. (1966). Microfluorometric characterization of intracellular nucleic acids and nucleoproteins by acridine orange. Acta Physiol. Scand..

[4] Sarnat H.B. (1985). L’acridine orange: un fluorochrome des acides nucléiques pour l’étude des cellules musculaires et nerveuses. Rev. Neurol..

