# Supplementary material for: Data on characterization of nano- and micro-structures resulting from glycine betaine surfactant/kappa-carrageenan interactions by Laser Scanning Confocal Microscopy and Transmission Electron Microscopy
Source: Data Brief. 2016 Sep 22;9:508–23. doi: 10.1016/j.dib.2016.09.026 (PMC5053038; doi:10.1016/j.dib.2016.09.026)
Supplement: Supplementary file 1 — Supplementary material [file mmc1.pdf]

Conficts of interests

We wish to confirm that there are no known conflicts of interest associated with this publication and there has been no significant financial support for this work that could have influenced its outcome.

Thierry BENVEGNU

12.09.2016
